# Pathological and physiological functions of presenilins

**DOI:** 10.1186/1750-1326-1-4

**Published:** 2006-06-12

**Authors:** Kulandaivelu S Vetrivel, Yun-wu Zhang, Huaxi Xu, Gopal Thinakaran

**Affiliations:** 1 Department of Neurobiology, Pharmacology and Physiology, The University of Chicago, Chicago, IL 60637, USA; 2 Center for Neuroscience and Aging, Burnham Institute for Medical Research, LaJolla, CA 92037, USA

## Abstract

Mutations in *PSEN1 *and *PSEN2 *genes account for the majority of cases of early-onset familial Alzheimer disease. Since the first prediction of a genetic link between *PSEN1 *and *PSEN2 *with Alzheimer's disease, many research groups from both academia and pharmaceutical industry have sought to unravel how pathogenic mutations in *PSEN *cause presenile dementia. *PSEN *genes encode polytopic membrane proteins termed presenilins (PS1 and PS2), which function as the catalytic subunit of γ-secretase, an intramembrane protease that has a wide spectrum of type I membrane protein substrates. Sequential cleavage of amyloid precursor protein by BACE and γ-secretase releases highly fibrillogenic β-amyloid peptides, which accumulate in the brains of aged individuals and patients with Alzheimer's disease. Familial Alzheimer's disease-associated presenilin variants are thought to exert their pathogenic function by selectively elevating the levels of highly amyloidogenic Aβ42 peptides. In addition to Alzheimer's disease, several recent studies have linked *PSEN1 *to familiar frontotemporal dementia. Here, we review the biology of PS1, its role in γ-secretase activity, and discuss recent developments in the cell biology of PS1 with respect to Alzheimer's disease pathogenesis.

## Background

In 1995 independent groups identified genetic linkage and mutations within *PSEN1 *(chromosome 14) and *PSEN2 *(chromosome 1) genes in several early onset familial Alzheimer's disease (FAD) kindreds [1-3]. Since then a number of research groups have focused on FAD-linked mutations and the biology of proteins encoded by these homologous genes. So far 140 and 10 mutations that co-segregate with FAD (in most cases before 60 years of age) have been identified in *PSEN1 *and *PSEN2*, respectively. *PSEN1 *and *PSEN2 *encode 467 and 448 amino acid-long polytopic transmembrane proteins, termed presenilin 1 (PS1) and presenilin 2 (PS2), respectively. The sequence identity between these two highly conserved proteins is greater than 65%. PS1 is expressed earlier than PS2 during mouse embryonic development indicating differential regulation of these proteins during development [4]. Although PS1 is relatively expressed at higher levels than PS2, both proteins are ubiquitously expressed in the brain and peripheral tissues in adult human and rodent.

Mutations in *PSEN1 *and *PSEN2 *are autosomal dominant, highly penetrant, and cause Alzheimer's disease (AD) symptoms before age 65, in some cases with onset of symptoms less than 30 years of age. Even though FAD-linked mutations in amyloid precursor protein (*APP*) and *PSEN *genes account for less than 5% of total AD cases, the phenocopies of these FAD mutations are reminiscent of late onset sporadic AD. As discussed below, PS1 and PS2 are subunits of a protein complex, termed γ-secretase, which cleaves several type I membrane proteins including APP within their transmembrane domain. In the case of APP, γ-secretase cleavage predominantly generates 39–43 amino acid-long peptides, termed β-amyloid peptides (Aβ), which accumulate in the brains of aged individuals and patients with AD. Although the mode of action (gain of function or loss of function) is still debated, FAD-linked mutations in PS selectively elevate the levels of highly amyloidogenic Aβ42 peptides by likely shifting the cleavage site in APP [5,6]. Nevertheless, FAD mutations may exhibit partial loss of function in some other physiological activities [7]. Because of its central role in the pathogenesis of AD, PS became the focus of much scrutiny and has been considered as a potential therapeutic target for the treatment of AD. More recently, novel *PSEN1 *mutations, as well as *PSEN1*-spliced variants, have been identified in several familiar frontotemporal dementia (FTD) kindreds, suggesting that PS1 may also play a role in FTD pathogenesis [8-12]. But the underlying mechanisms are still elusive. In addition, several studies have identified physiological functions of PS1 beyond AD and FTD including apoptosis, calcium homeostasis, neurite outgrowth, and synaptic plasticity [reviewed in [13]]. In this chapter, we mainly focus on recent advances in the cell biology of PS, and discuss the function of PS as it relates to AD.

### PS topology

**Figure 1:**
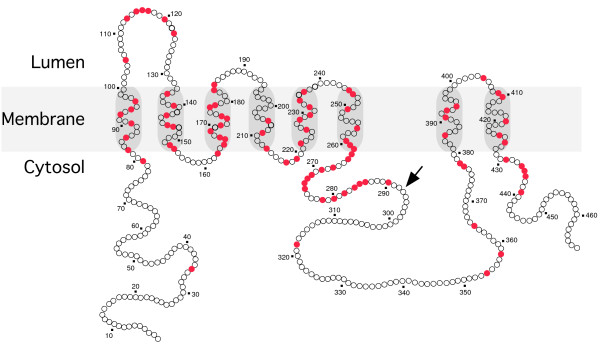
**Schematic representation of PS1 structure**. In this model, PS1 is depicted to traverse the membrane eight times. Each circle represents an amino acid, and shaded circles represent individual residues mutated in FAD cases. In addition to point mutations, two deletion mutations ⊗I83/M84 and ⊗S290–S319 have been identified. Arrow marks the major site of endoproteolytic cleavage, which occurs when nascent PS1 molecule assembles with nicastrin, APH-1 and PEN-2.

Determination of PS protein topology is of particular interest because it may facilitate a better understanding of the structural and functional relationship of γ-secretase activity. The transmembrane (TM) topology of PS1 is still being debated, though several topology models have been proposed based on different experimental approaches. Most of these studies use antibodies, engineered N-glycosylation acceptor sites, protease digestion, and gene fusions with reporters, to map the cytosolic or lumen regions of the protein. The amino acid sequence of PS contains ten hydrophobic regions (HR) that can potentially function as TM domains, leading to the proposal of several models for PS with 6 to 9 TM segments. Nevertheless, an eight TM topology model with N- and C-termini, and a hydrophilic loop domain between TM 6 and 7 facing the cytosol, has been widely accepted [14] (Fig. 1). While there is uniform agreement between various models in cytosolic location of the N-terminus and the TM assignments of the first six HR, the models differ with respect to HR seven through ten as membrane spanning domains; also, the location of PS1 C-terminus has long been debated as well. A recent study using glycosylation acceptor sequences also confirmed the luminal orientation of the first hydrophilic loop and cytosolic orientation of N-terminus and a large hydrophilic loop (between TM 6 and 7), which are in agreement with the eight TM model [15]. However, in contrast to the eight TM model, the C-terminus was found oriented to the lumen suggesting that an HR near the C-terminus functions as the ninth TM segment.

### Endoproteolysis of PS

PS1 is synthesized as a 42- to 43-kD polypeptide that undergoes highly regulated endoproteolytic processing within the large cytoplasmic loop domain connecting putative TM segments 6 and 7 to generate stable 27- to 28-kD N-terminal (NTF) and 16- to 17-kD C-terminal (CTF) fragments [16] by an uncharacterized proteolytic activity. Endoproteolytic processing of PS is a highly conserved and, perhaps, a critical event that regulates the stability of PS1 and possibly the biological activity of PS. While full length PS1 is relatively short-lived with a half-life of 1–2 h, endoproteolytically processed derivatives (NTF and CTF) have a half-life of 24 h [17,18]. Moreover, the endoproteolytic event has been identified as the activation step in the process of PS1 maturation as it assembles with other γ-secretase subunits nicastrin, APH-1, and PEN-2 (described below) [19]. Exception to this initial activation event is the maturation of FAD-associated PS1ΔE9 variant, which lacks a region including the endoproteolytic cleavage site encoded by exon 9 (Fig. 1). PS1ΔE9 holoprotein is metabolically stable, forms a complex with other γ-secretase subunits and generates functional γ-secretase activity.

In transfected cells only a fraction of overexpressed PS1 is converted to stable NTF and CTF, whereas the vast majority of nascent PS1 holoprotein is highly unstable and is rapidly degraded. The process of PS1 endoproteolysis is a tightly regulated and saturable event, with PS1 NTF and CTF accumulating in 1:1 stoichiometry [16,20]. Transgenic overexpression of human PS1 in mice replaces endogenous mouse PS1 by a highly selective and compensatory mechanism, and the extent of replacement is proportional to the level of exogenous PS1, which suggests that exogenous and endogenous PS compete for limiting cellular factors [16,20]. In cells co-expressing PS1 and PS2, the assemblies consist of either of PS1 derivatives or PS2 derivatives but not mixed assemblies (for example, PS1NTF•PS2CTF). However, expression of chimeric PS1/PS2 holoprotein is endoproteolysed and forms heteromeric assemblies made of PS1NTF•PS2CTF [21]. Furthermore, exogenous PS1 NTF does not co-assemble with endogenous PS1 CTF. Nevertheless, co-expression of exogenous NTF and CTF can reconstitute functional γ-secretase in PS-deficient cells [22,23]. These findings indicate that association between N- and C-terminal domains of PS1 holoprotein occurs prior to endoproteolysis; and following assembly, PS-derived NTF/CTF do not exchange between γ-secretase complexes containing either PS1 or PS2.

Experimental deletion and replacement mutants and domain swap experiments have been useful in identifying amino acid residues that are critical for PS1 endoproteolysis as well as γ-secretase activity. Deletions and substitutions near the putative endoproteolytic cleavage sites between Thr291 and Ala299 [17] abolished PS1 endoproteolysis, but the resulting stable holoprotein still retained γ-secretase activity [24]. In contrast, aspartate residues at position D275 (in TM6) and D385 (in TM7) of PS1 are critical for both PS1 endoproteolysis and γ-secretase function [25]. Furthermore, residues critical for both PS1 endoproteolysis and γ-secretase activity have been identified within TM1 of PS1 [26]. These findings have been inconclusive with reference to the correspondence between PS endoproteolysis and γ-secretase activities. With a few exceptions, well-characterized and highly potent γ-secretase inhibitors do not affect PS1 endoproteolysis, providing more definite proof that PS endoproteolysis and γ-secretase activity is pharmacologically distinct [27]. The enzyme activity responsible for PS1 endoproteolysis has not been identified. Based on the available data one cannot exclude the possibility that PS1 endoproteolysis may be an autocatalytic event, which occurs during the maturation of unstable nascent PS1 holoprotein into stable derivatives. Clues from the deletion of domains far from the endoproteolytic site are consistent with a potential conformational change associated with the process of PS1 endoproteolysis and maturation. For example, deletion of TM1 through TM2 (PS1ΔM12) resulted in an endoproteolysis-defective mutant, which unlike PS1 holoprotein is extremely stable [28,29]. Interestingly, a large hydrophilic loop domain connecting the TM domain 6 and 7 (residues 304–371) of PS1, which includes a caspase cleavage site (D345) and serves as the interaction domain for several PS-associated proteins, is dispensable for PS1 endoproteolysis and γ-secretase activity [30].

### PS-dependent γ-secretase is involved in intramembranous proteolysis of type I membrane proteins

In the past few years there has been accumulating interest in understanding regulated cleavage of type I membrane proteins within their transmembrane domains. Aβ40 and Aβ42 peptides are released from APP by intramembranous cleavage at two major sites within the TM. In keeping with the nomenclature of previously described α- and β-secretase cleavage of APP within the extracellular domain, the intramembranous cleavage of APP was termed the "γ-secretase" cleavage. The realization that cleavage at one of the sites that corresponds to +42 residue of Aβ is enhanced 2-fold in FAD cases [6] led to increased scrutiny of this cleavage process. De Strooper and colleagues [31] first reported a direct role for PS1 in this process when they noted loss of Aβ secretion and accumulation of APP C-terminal fragments (CTF) in *PS1*^-/- ^neurons. Soon after, a striking similarity between PS-mediated cleavage of APP and the proteolytic cleavage of transmembrane Notch receptor began to emerge [32-34]. In both cases, membrane tethered CTF, rather than the full-length proteins, serves as the substrates for γ-secretase cleavage. Struhl and Adachi [35] systematically examined the requirements for PS1/γ-secretase cleavage and concluded that PS1 can mediate sequence-independent cleavage of a diverse set of transmembrane proteins with short extracellular domains. Consistent with APP and Notch processing, cleavage efficiency of experimental proteins was inversely proportional to the length of the extracellular domain of the proteins [35].

**Figure 2:**
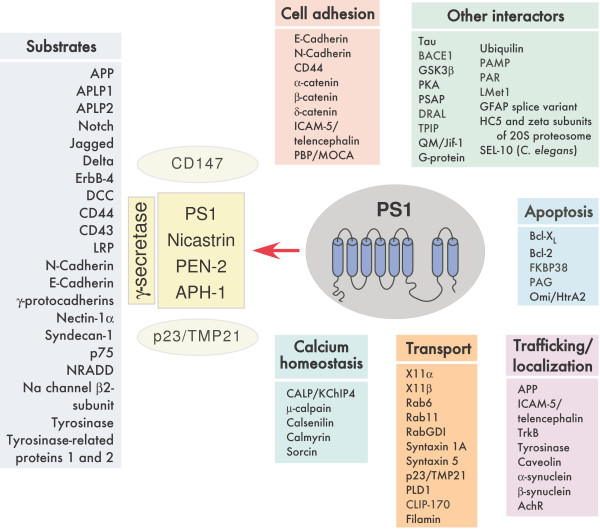
**Multifaceted functions of PS1 mediated through the interacting proteins**. The figure represents the multiple roles of PS1 in cellular functions. PS1 associates with nicastrin, APH-1 and PEN-2 to form the γ-secretase, which cleaves a set of type I membrane protein substrates including APP and Notch. Transmembrane proteins CD147 and p23/TMP21 have been shown to interact with γ-secretase complex and modulate Aβ production. In addition, PS1 interacts with several proteins that are involved in important physiological functions such as calcium homeostasis, vesicular transport, cell adhesion, etc. Trafficking of certain membrane proteins, including some γ-secretase substrates also seems to be influenced by PS1 deficiency, inhibition of γ-secretase activity, or expression of FAD-linked PS1 variants.

As expected from the relaxed sequence specificity of γ-secretase, a wide range of type I transmembrane substrates has been described within the last few years, extending a physiological role for PS1 beyond the nervous system and AD (Fig. 2). These include homologues of APP (APLP1 and APLP2) [31,36], Notch1 homologues [32-34], Notch ligands Delta and Jagged [37], the receptor tyrosine kinase ErbB-4 [38], cell surface adhesion protein CD44 [39], the mucin-type molecule CD43 [40], low-density lipoprotein receptor-related protein [41], cell adhesion receptors N- and E-cadherins [42,43], cadherin-related gamma-protocadherins [44], synaptic adhesion protein nectin-1α [45], netrin receptor DCC [46], cell surface heparin sulphate proteoglycan syndecan-3 [47], p75 neurotrophin receptor [48] and its homolog NRADD [49], the voltage-gated sodium channel β2-subunit [50], tyrosinase and tyrosinase-related proteins 1 and 2 [7] etc. As with the case of APP and Notch1, γ-secretase cleavage of these additional substrates is preceded by cleavage(s) within their extracellular domain. In several cases PS1/γ-secretase cleavage releases an intracellular domain analogous to Notch intracellular domain that can translocate into the nucleus. It remains to be determined whether intracellular domains of all γ-secretase substrates engage in signal transduction upon gaining entry into the nucleus. For example, γ-secretase cleavage of DCC terminates the cAMP/PKA signaling cascade and potentially modulates glutamatergic synaptic transmission in neurons [51].

### PS1 stability and functions in γ-secretase require other components

As described above, overexpression of PS1 alone neither substantially increases the levels of mature PS1 derivatives nor elevates Aβ generation in cultured cell lines and transgenic mice. Based on these findings, a model was proposed wherein the abundance of mature PS1 fragments is regulated by interaction with limiting cellular factor(s) [20]. Genetic and biochemical approaches have identified three additional proteins that are crucial for the stability of PS1 as well as γ-secretase activity. Goutte and colleagues used genetic screens to identify two genes, named *aph-1 *and *aph-2 *(anterior pharynx defective), which act in the GLP-1/Notch pathway during the early stages of *C. elegans *embryogenesis [52,53]. A type I transmembrane protein termed Nicastrin, which is the mammalian homolog of *aph-2*, was identified simultaneously by biochemical methods as a protein bound to PS1 [54]. An independent *C. elegans *genetic screen identified *aph-1 *and *pen-2 *as enhancers of sel-12/Presenilin function, and showed that *aph-2*, *aph-1 *and *pen-2 *are required for Notch signaling, PS1 accumulation, and γ-secretase activity [55]. APH-1 is a multipass transmembrane protein encoded by two (human) or three (rodent) genes that can be alternatively spliced. PEN-2 is a protein with two transmembrane domains encoded by one gene. It is now clear that PS, nicastrin, APH-1, and PEN-2 are stoichiometric components of high molecular weight γ-secretase complex. Co-expression of these four transmembrane proteins is sufficient to reconstitute γ-secretase activity in yeast, which lacks these mammalian orthologs [56]. Furthermore, co-expression of all four proteins is sufficient to overcome the limitation in generating excess PS-derived NTF and CTF in transfected cells [57-59]. Thus, PS1 endoproteolysis, stability and accumulation of PS1 NTF/CTF are regulated by the availability of stoichiometric levels of nicastrin, APH-1 and PEN-2. Most interestingly, biogenesis, maturation, stability, and the steady state levels of γ-secretase components are co-dependent. Down regulation or targeted gene disruption of any one of these components affects maturation and stability of other subunits, indicating that their assembly into a high molecular weight complex is a highly regulated process that occurs during biosynthesis of these polypeptides. For example, the heavily glycosylated type I membrane protein nicastrin does not mature and exit the ER in cells lacking PS1 expression [60]. PS1 deficiency also leads to altered intracellular trafficking of PEN-2 and APH-1, as well as their destabilization [61,62]. On the other hand, PS1 fails to undergo endoproteolysis to generate stable NTF and CTF in nicastrin^-/- ^cells or when PEN-2 is downregulated by RNAi [62-64].

The sequence of events in the assembly of the γ-secretase complex is only beginning to unravel at this time. Available evidence supports the formation of an early intermediate sub-complex made of APH-1 and nicastrin [65]. The proximal C-terminus region PS1 holoprotein then binds to this subcomplex by interacting with nicastrin TM domain [66]. During the final step in the assembly, PEN-2 associates with the complex by interaction with TM 4 of PS1 2 [67,68], concurrent with PS1 endoproteolysis. The observation that membrane-permeable γ-secretase inhibitors markedly increase cell surface levels of PS1 and PEN-2 without affecting that of nicastrin also implies a tighter association between PS1 and PEN-2 [61]. Despite the identification of the core components, the precise stoichiometry of the active γ-secretase complex remains obscure. First, the existence of distinct subsets of γ-secretase complexes consisting of different PS or APH-1 subunits has been reported from studies conducted in cell culture and animal models [69,70]. Second, a recent study demonstrated the existence of PS dimer at the core of the γ-secretase complex with the substrates being processed between the PS monomers [71]. In addition, the use of different biochemical purification methods and detergents has led to the apparent size discrepancy of active γ-secretase complexes, with estimates ranging from 250 kDa [58] to 2 MDa [72].

### PS1 functions as the catalytic center of γ-secretase

The exact functional contribution of each γ-secretase subunit to enzyme activity still remains elusive. Two lines of investigation strongly support the notion that PS1 may form the catalytic center of γ-secretase. First, Wolfe and colleagues identified the presence of two highly conserved aspartate residues in transmembrane domains 6 (D257 in PS1 and D263 in PS2) and 7 (D385 in PS1 and D366 in PS2) that are indispensable for γ-secretase activity, and suggested that γ-secretase is a transmembrane aspartyl protease [25]. Second, it was found that highly specific transition-state analogue inhibitors of γ-secretase specifically bound to PS1 NTF/CTF heterodimer [73]. As discussed above, the stoichiometry of γ-secretase subunits has not been firmly established. A recent study demonstrated the existence of PS dimer at the core of γ-secretase complex and proposed a model where the substrates are processed between the PS monomers [71]. Interestingly, APP and Notch can interact with PS1 in the presence of active site γ-secretase inhibitors, revealing the presence of a docking site within PS1 that is distinct from the catalytic site [74,75]. In a recent study Yu and colleagues reported that the extracellular domain of nicastrin interacts with ectodomain cleaved APP and Notch, and thus functions as the γ-secretase substrate receptor [76]. A recent study suggested that a widely expressed cell surface type I transmembrane glycoprotein CD147 (also called basigin and EMMPRIN) may function as a regulatory subunit of the γ-secretase complex [77]. Downregulation of CD147 expression caused a modest increase in Aβ production. Interestingly, unlike the integral γ-secretase components, overexpression or downregulation of CD147 did not affect the stability of γ-secretase complex [77]. Similarly, a recent study reported that p23/TMP21, a member of the p24 family of transmembrane proteins involved in vesicle trafficking between the ER and Golgi apparatus binds to γ-secretase complex. Knockdown of p23/TMP21 expression did not affect the steady-state levels of γ-secretase subunits, but resulted in increased generation of Aβ40 and Aβ42 peptides without affecting ε-cleavage of APP or Notch [78]. Despite these recent advances, it is still intriguing how FAD-associated point mutations that are widely scattered throughout the protein, many of which are located far away from the putative catalytic site, selectively alter the cleavage site preference in APP in a manner that increases Aβ42 production.

### Subcellular localization of PS1

PS1 has been localized to multiple organelles including the endoplasmic reticulum (ER), ER/Golgi intermediate compartments, Golgi apparatus, endosomes, lysosomes, phagosomes, plasma membrane, and mitochondria. Quantitative immunoelectron microscopy estimates of endogenous PS1 in CHO cells showed that the vast majority (52%) of PS1 is present in pre-Golgi membranes that include nuclear envelope, ER and vesicular-tubular clusters that are positive for COP1, whereas only about 1% of total label was localized in the Golgi complex [79]. A significant amount of PS1 (25%) was localized in the plasma membrane and 13% in endocytic compartments. However, a recent biochemical study estimated that only 6% of PS1 and γ-secretase activity exists at the cell surface [80]. Consistent with the later estimates, cell fractionation studies show that the majority of the mature components of endogenous γ-secretase complex are present in intracellular organelles [81]. Furthermore, non-ionic detergent extraction revealed the presence of presenilin and other γ-secretase subunits in cholesterol- and sphingolipid-rich, detergent-resistant membrane microdomains of post-Golgi, TGN and endosome membranes. Association of γ-secretase components with detergent-resistant membranes is sensitive to cholesterol depletion, fulfilling a stringent criterion expected of *bona fide *lipid raft associated proteins. Using magnetic immunoisolation, active and mature components of γ-secretase complex were found to co-reside in lipid raft microdomains with VAMP-4 (TGN), syntaxin 6 (TGN vesicles) and syntaxin 13 (late endosomes). Interestingly, the cell-surface raft-associated protein SNAP-23 does not co-reside with γ-secretase subunits, further validating intracellular compartmentalization of γ-secretase complex [81].

### Differential functions of wild type and FAD-associated PS1 and PS2

Over 130 mutations (>258 families) in *PSEN1 *and 9 mutations (15 families) in *PSEN2 *have been identified as autosomal dominant, highly penetrant mutations that cause early-onset AD. In general, mutations in *PSEN2 *are associated with a later age of onset compared to *PSEN1 *(mean familial age of onset 57.1 years and 44.1 years, respectively) and cause slower disease progression [82]. FAD-linked mutations in PS intriguingly influence γ-secretase cleavage by an elusive mechanism that modulates the proteolysis of APP to selectively enhance the generation of highly amyloidogenic Aβ42 peptides [5,6]. However, the high frequency of FAD-associated mutations in *PSEN1 *compared to *PSEN2 *may be an indicator of differential functions of γ-secretase containing PS1 or PS2. Indeed, *in vivo *complementation of PS1 deficiency with PS2 in transgenic mice indicates functionally different roles for PS1 and PS2 [70]. In the absence of PS1, transgenic expression of wild type PS2 neither increases Aβ40/42 nor rescues Notch-associated skeletal defects in embryos. However, complementation of FAD-associated variants of PS2 selectively elevated Aβ42 levels and rescued Notch-associated defects. These studies provide compelling support for the existence of functionally distinct γ-secretase complexes, and further support the idea of gain of function by FAD-linked PS variants.

Precisely how FAD-linked PS mutations influence Aβ42 production is still not understood. Altered conformation has been suggested as a mechanism by which FAD-associated PS variants may influence specificity of cleavage site within APP, thus elevating Aβ42 production. For example, it has been shown that FAD-associated mutations change the proximity of N- and C-termini of PS1 [83]. On the other hand, non-steroidal anti-inflammatory drugs such as sulindac sulfide, ibuprofen, indomethacin and flurbiprofen, which specifically lower Aβ42 levels without affecting Aβ40 production [84], decrease the proximity of PS1 N- and C-terminus and PS1 to APP C-terminus [85]. Nevertheless, how these proximity measures of FAD mutants relate to conformational changes in the catalytic site or the substrate-docking site of γ-secretase in a manner that fosters elevated Aβ42 production remains to be understood. Recent studies also suggest that the adverse property of FAD-linked PS alleles is not limited to altering cleavage site specificity of substrates APP and Notch. For example, large-scale gene expression profiling in brains of conditional PS1 knockout mice and transgenic mice expressing wild type or FAD-linked mutant PS1 suggests that the FAD-linked PS1 variant produces transcriptome changes primarily by gain of aberrant function [86]. Furthermore, the expression and activity of neprilysin, an Aβ degrading enzyme, is regulated by γ-secretase activity. *In vitro *studies show that transcription from neprilysin gene promoter can be activated by cytosolic domains released from APP, APLP1 or APLP2 by γ-secretase cleavage, and FAD-associated PS1 mutations increase neprilysin levels in brains of patients with mutant PS1 alleles [87]. Finally, since Aβ42 activates neutral sphingomyelinase and Aβ40 inhibits hydroxymethyl-CoA reductase, an indirect role for PS in maintaining cholesterol and sphingomyelin levels through Aβ40 and Aβ42 production has been proposed [88]. As expected from increased production of Aβ42, FAD-associated PS1 mutants specifically increase cellular cholesterol and decrease sphingomyelin levels.

In addition to causing a shift in APP cleavage site, FAD-associated PS1 mutations may show partial loss of function in some other physiological activities. PS1 has been suggested as being involved in not only Aβ generation but also other processes leading to neurodegeneration. Tanemura et al. showed that tau was hyperphosphorylated in FAD-associated PS1 mutant (I213T) knock-in mice in the absence of Aβ deposition [89], whereas neurodegeneration of PS conditional double knockout mice is associated with tau hyperphosphorylation [90]. Part of the underlying mechanisms might be that PS modulates PI3K/Akt and ERK signaling pathways to downregulate GSK-3 activity and tau phosphorylation; and FAD mutations inhibit these pathways in a way similar to that by PS1 deficiency [91,92]. Moreover, a recent study by Wang et al. found that melanin synthesis in FAD-associated PS1 M146V knock-in mice was impaired, which was consistent with their observation that γ-secretase inhibitor blocked melanin synthesis [7].

### PS1 regulates trafficking of select membrane proteins

In addition to its function as the catalytic subunit of γ-secretase, PS directly or indirectly regulates the trafficking of select membrane proteins. As mentioned above, PS1 deficiency resulted in abnormal intracellular trafficking of the other three γ-secretase components, nicastrin, APH-1 and PEN-2. In addition, PS1 deficiency in neurons accelerated the secretion of α- and β-secretase cleaved APP ectodomain [93]. Further analysis revealed that PS1 regulates biosynthetic secretory trafficking of APP. Absence of PS1 or the expression of a loss of function PS1 variant resulted in increased budding/generation of APP-containing vesicles from both ER and TGN with a concomitant increase in complex glycosylation and cell surface appearance of APP [29,94]. In addition, the half-life and steady-state residence of full length APP and APP CTFs at the cell surface were greatly increased [95,96]. Interestingly, FAD-linked PS1 variants significantly reduced budding of APP-containing vesicles from both ER and TGN, resulting in decreased delivery of APP to the cell surface [94]. These findings raised the possibility that FAD-linked PS1 variants may influence APP processing by increasing the time of APP residing at the TGN, consequently prolonging their availability for cleavage by β- and γ-secretases within the TGN. Direct evidence to support a trafficking role of PS1, independent of its function in γ-secretase activity, emerged from analysis of membrane proteins that are not substrates for γ-secretase cleavage. For example, complex oligosaccharide modification and brain-derived neurotrophic factor-induced phosphorylation of the neurotrophin receptor TrkB was severely affected in neurons lacking PS1 expression [93]. Furthermore, neuron specific intercellular adhesion molecule (ICAM-5)/telencephalin accumulated intracellularly in autophagic vacuoles and displayed extended half-life in the absence of PS1 [97]. Similarly, α- and β-synuclein were found mislocalized in autophagic organelles in PS1-deficient neurons but not in neurons treated with selective γ-secretase inhibitors [98]. Remarkably, increased assembly and cell surface delivery of nicotinic acetylcholine receptors (a mutimeric polytopic membrane protein complex) was observed in cells expressing a loss of function PS1 mutant that exhibited accelerated cell surface delivery of APP and slower kinetics of Aβ secretion [29]. While a role for PS1 in regulating protein trafficking remains attractive, the growing number of γ-secretase substrates raises the possibility of impaired turnover of CTFs derived from type I membrane proteins indirectly influencing protein trafficking in cells lacking PS1 expression or γ-secretase activity. Indeed, a recent study demonstrated that PS deficiency resulted in mislocalization of post-Golgi tyrosinase-containing vesicles; and such an abnormal trafficking of tyrosinase is γ-secretase-dependent and accompanied by simultaneous accumulation of its C-terminal fragment [7]. On the other hand, there is a possibility that PS1 indirectly regulates protein trafficking via its interaction with protein trafficking factors. For example, PS1 has been shown to interact with small factors such as Rab11, Rab6 and Rab GDI that are involved in regulation of vesicular transport [99-101]; and modulation of Rab6-mediated transport has been shown to affect APP processing [102]. Recently Cai et al. also showed that PS1 interacted with phospholipase D1 (PLD1), a phospholipid-modifying enzyme that regulates membrane trafficking events [103,104]. The results demonstrated that this interaction recruited PLD1 to the Golgi/trans-Golgi network and thus possibly modulated APP trafficking, since overexpression of PLD1 promoted generation of APP-containing vesicles from the TGN.

### PS-interacting proteins

Over the past several years many investigators employed yeast two-hybrid assays and candidate approaches to identify proteins that interact with various domains of PS [105] (Fig. 2). As an outcome, several PS interacting proteins have been identified, including members of a family of armadillo-related proteins such as β-catenin; cell surface transmembrane protein E-cadherin; neuronal cell adhesion molecule telencephalin; filamin, an actin binding protein; PBP/MOCA, a protein with limited homology to Dock180; the enzyme glycogen synthase kinase-3β microtubule-associated protein tau; calcium binding proteins such as calsenilin, calmyrin, sorcin, mu-calpain and CALP/KChIP4; anti-apoptotic molecule Bcl-X_L_; Rab11 and Rab6, small GTPases involved in regulation of vesicular transport; RabGDI, a regulatory factor in vesicular transport; PLD1, a phospholipid-modifying enzyme involved in membrane trafficking events; syntaxin 1A, a t-SNARE localized in the synaptic plasma membrane; syntaxin 5, a t-SNARE that mediates transport between the ER and Golgi; adaptor proteins X11α and X11β CLIP-170, the microtubule plus-end-tracking protein; brain G-protein, G_o_; Ubiquilin, a protein containing ubiquitin-related domains; HC5 and ZETA subunits of the catalytic 20S proteasome; TPIP, a tetratricopeptide repeat containing protein; PAG, a protein of the thioredoxin peroxidase family, PSAP, a PDZ-like protein, QM/Jif-1, a negative regulator of c-Jun; DRAL, an LIM-domain protein; proliferation-associated gene product, a protein of the thioredoxin peroxidase family; β-secretase, BACE1; PAMP and PARL, two novel putative metalloproteases; Omi/HtrA2, a serine protease involved in the mammalian cellular stress response; Met1, a novel putative methyltransferase; mitochondrial immunophilin FKBP38; a splice variant of glial fibrillary acidic protein, etc. The *C. elegans *PS homologue SEL-12 was recently reported to interact with SEL-10, a Cdc4p-related protein. In many instances the physiological role for the identified interaction between the putative protein with PS1 or PS2 is not clearly defined. For example, the PS interaction with the anti-apoptotic Bcl2 family member Bcl-X_L _offers a potential mechanism by which PSs might regulate apoptosis [106]. However, it is unclear at present whether an increased apoptotic response associated with the expression of FAD-linked PS variants noted in several studies can be attributed to differential interaction of mutant PS1 or PS2 with Bcl-X_L_. Similarly, the absence of PS1 or the expression of FAD-linked PS1 mutant results in increased glycogen synthase kinase-3β activity, enhanced kinesin light chain phosphorylation, and concomitant reduction in kinesin-based axonal transport [107]. But it remains unclear whether PS1• glycogen synthase kinase-3β interaction plays a direct role in regulating kinesin-based axonal transport. Finally, it is somewhat puzzling that neither regulated metabolism of PS nor the enhanced production of Aβ42 by FAD mutants appears to be influenced by any of the reported PS interacting proteins.

## Conclusion

Mutations in *PSEN1 *and *PSEN2 *genes co-segregate with early onset FAD cases and genetic ablation of these genes eliminates Aβ production in transgenic mice. Replacement of conserved aspartate residues in TM 6 and 7 of PS resulted in inactivation of γ-secretase activity and PS1 fragments were specifically photolabeled by transition state analogues of γ-secretase. Taken together these studies strongly support PS as catalytic subunit of γ-secretase and γ-secretase as transmembrane aspartyl protease. Abolishment of Aβ production by inhibiting γ-secretase is therefore considered a potential therapeutic strategy for the treatment of AD and several pharmacological inhibitors, which specifically target processed PS fragments, have been developed. However, further studies have unraveled several other functionally important substrates of PS-mediated γ-secretase cleavage including the type I membrane protein Notch that is critical for development. These studies indicate that generalized inhibition of γ-secretase could potentially result in severe consequences by interfering in other physiological and developmental processes. It will be more reasonable to develop inhibitors that specifically target proteolytic processing of APP but not other substrates by PS/γ-secretase, which requires logical design and high throughput screening. Recent studies suggest the use of separate active sites by γ-secretase in the proteolytic processing of APP and Notch. Further, APP and Notch processing have been shown to occur in spatially distinct sites. Also interesting is the existence of tissue specific and compositionally distinct γ-secretase complexes. Therefore, development of specific inhibitors targeting the active site of γ-sceretase involved in APP processing or targeting the γ-secretase localized in subcellular membrane microdomain involved in APP processing is promising as a novel therapeutic strategy. The use of non-steroidal drugs, NSAIDs, has been shown to be effective in specifically reducing the levels of Aβ42 peptides without affecting Aβ40 production and Notch processing. The mechanism of FAD-associated PS variants in elevating levels of Aβ42 is still being explored. Altered conformation and proximity of PS1-derived fragments themselves or with the substrate APP were shown to be the possible cause for the pathogenic functions of FAD-associated PS1 variants. It is still intriguing how widely scattered FAD-associated PS1 mutants have the same functional effect on APP processing. PS1 has been shown to be involved in the subcellular trafficking of APP and FAD-linked PS1 variants increase the retention of substrate in compartments where γ-secretase was localized. Experimental mutations are quite useful in identifying the functions of the individual domains of PS1. Transgenic mice with conditional ablation of PS1 and expression of FAD-associated human PS variants have provided some clues about the physiological consequences and stages in the development of disease. In contrast to PS1, not much information is available on the functions of PS2 or PS2-mediated γ-secretase complex. Most of the aggressive forms of FAD cases (less than 30 years of age at onset) are associated with PS1 rather than PS2. The lesser frequency of FAD-linked PS2 mutations compared to that of PS1 also suggests a redundant role for PS2 containing γ-secretase complexes. But interestingly, FAD-associated variant PS2N141I is able to rescue Notch-associated developmental defects compared to PS2 wt in the absence of PS1, which makes the precise functions of different PS/γ-secretase complexes perplexing. On the other hand, several PS interacting proteins have been identified but the functional relevance of such interactions is still not completely elucidated. Since the first clue for PS association in FAD was identified a decade ago, we have gained more insights into these novel proteins. Nonetheless with the information we gained on these proteins, finding a cure for the devastating AD is still promising.
